# Development of Ferromagnetic Superspins in Bare Cu Nanoparticles by Electronic Charge Redistribution

**DOI:** 10.3390/ijms161023165

**Published:** 2015-09-24

**Authors:** Erdembayalag Batsaikhan, Yen-Cheng Chen, Chi-Hung Lee, Hsiao-Chi Li, Wen-Hsien Li

**Affiliations:** Department of Physics, National Central University, Jhongli 32001, Taiwan; E-Mails: erdembaylag@yahoo.com (E.B.); yencheng0509@gmail.com (Y.-C.C.); 992402003@cc.ncu.edu.tw (C.-H.L.); lc205063719710096@rocketmail.com (H.-C.L.)

**Keywords:** Cu nanoparticle, ferromagnetic superspin, electronic charge density

## Abstract

We report on the results of investigating the ferromagnetic properties of bare Cu nanoparticles. Three sets of bare Cu nanoparticle assemblies with mean particle diameters of 6.6, 8.1, and 11.1 nm were fabricated, employing the gas condensation method. Curie-Weiss paramagnetic responses to a weak driving magnetic field were detected, showing the appearance of particle superspins that overcomes the diamagnetic responses from the inner core. The isothermal magnetization displays a Langevin field profile together with magnetic hysteresis appearing even at 300 K, demonstrating the existence of ferromagnetic superspins in the Cu nanoparticles. Shifting of a noticeable amount of electronic charge from being distributed near the lattice sites in bulk form toward their neighboring ions in nanoparticles was found. The extended 3*d* and 4*s* band mixture are the main sources for the development of localized 3*d* holes for the development of ferromagnetic particle superspins in Cu nanoparticles.

## 1. Introduction

Copper is one of the most widely used materials of great importance in many industrial applications, especially in the electrical sector owning to its high conductivity, rich abundance, and low cost. Size reduction of materials for higher efficiency or new device functionality is one of the most focused subjects of current research [1,2]. Copper in its nano-sized form has attracted considerable attention owing to its novel catalytic, optical, and electrical properties [3,4,5], as well as its practical applications as environment remediation, as antibacterial agents, and as heat exchangers [6,7,8,9,10]. Understanding copper nanoparticles (NPs) is essential to understanding Cu-based nano-devices. A NP consists of a limited number of atoms, with a large fraction of them being on the surface. It is known that the physical properties of NPs will be affected by symmetry breaking at the surface [11], by disruption of lattice periodicity at the surface [12,13,14], and by quantum confinement of the conduction electrons [15,16,17]. Each of these effects can significantly alter the electronic and magnetic behaviors. A great variety of magnetic phenomena, such as giant paramagnetism, superparamagnetism, and even spontaneous magnetism have all been observed in various NPs [18,19,20,21,22,23,24,25,26].

In its bulk form Cu is known to be weakly diamagnetic, driven by the Lenz diamagnetic responses from the completely filled ions core and 3*d*-band [27]. This situation can be altered when the size of the system is reduced to the nanometer scale. Ferromagnetic behavior in thiol-capped Cu NPs and superparamagnetic character in amine-capped Cu NPs have been reported [6]. There are at least two effects that may affect the magnetic properties of Cu when it is reduced to NP. First, the transfer of charges from the surface atoms to the inner ones has been found to be energetically favorable to stabilizing the core for nano-sized particles [28,29]. Second, the image charges of the surface electrons, known as Fermi holes [30,31], may result in imbalanced spins near the surface [32]. As a result, the number of uncompensated spins in the core, as well as on the surface, can increase noticeably which, in turn, can give rise to a non-zero magnetic moment for the NP.

Until recently, most of the studies made on the magnetic behavior of Cu NPs were performed using polymer-capped particles, rarely with bare NPs. The physical behavior of capped NPs, driven by the dominating interactions between the NPs and the capping agents, can be very different from their bare form. In this article, we report on the observation of ferromagnetic spin polarization in capping-free Cu NPs. Here, we emphasize on the changes in magnetic properties originating from the size effects, while avoiding the complications that may arise from the capping agents. The differences in the electron density distributions between bulk and nano-sized Cu are used to understand the observations.

## 2. Results and Discussion

### 2.1. Sample Fabrication and Characterization

**Figure 1:**
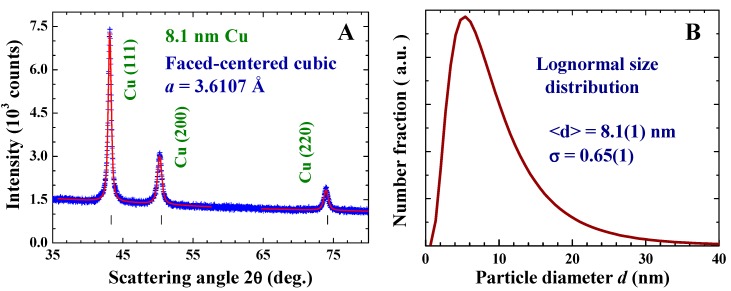
(**A**) X-ray diffraction pattern of the Cu nanoparticle assembly at 300 K, revealing a face-centered cubic crystalline structure. The solid curves indicate the calculated profiles of the diffraction peaks, assuming a lognormal size distribution with a center at 8.1 nm and a standard deviation width of 0.65; and (**B**) size distributions obtained from the X-ray diffraction profile.

Three sets of Cu nanoparticles were fabricated by employing the gas condensation method [26], where the mean particle size and size distribution were controlled by the proper choices of chamber pressure and source temperature. High-purity Cu spheres (99.99% pure and 2 mm in diameter) were heated by a current source (55/65/80 A) and were evaporated at a rate of 0.05 Å/s in an Ar atmosphere under pressure of 2 torr. The evaporated particles were collected on a non-magnetic SS316 stainless steel plate placed 20 cm above the evaporation source and maintained at 77 K. After restoration to room temperature, the NPs, which were only loosely attached to the collector, were stripped off. The samples thus fabricated were in powdered form, consisting of macroscopic amounts of individual Cu NPs. The resultant powders were no longer yellow but dark black, indicating that the absorption bands of the powders had blue-shifted to the invisible region, as is the cases with most metal NPs. It appeared that the samples were sensitive to exposure to the air. The samples used in the present measurements were stripped off from the collector and loaded into the capsulated sample holders in an Ar atmosphere enclosed in the chamber after evaporation. They were kept in a vacuum afterward at all times. The X-ray diffraction patterns of all three sets of Cu NP powders can be associated with a face-centered cubic (fcc) Cu structure. No traces of oxidation phases or elements other than Cu may be identified from the diffraction patterns. Figure 1A shows the X-ray diffraction pattern of the representative Cu NPs fabricated using a current source of 65 A, taken at room temperature. As expected, the diffraction peaks appear to be much broader than the instrumental resolution, reflecting the broadening of peak profiles due to the finite-size effect. The mean particle diameters are determined by fitting the diffraction peaks to the diffraction profiles of finite sized particles [26]. The solid curves in Figure 1A indicate the calculated pattern assuming a log-normal size distribution (Figure 1B) with a center at 8.1 nm and a standard deviation of 0.65. The mean particle diameters determined for the Cu NPs fabricated using current sources of 55 and 80 A are 6.6 and 11.1 nm, respectively. The fcc lattice constant that we obtained for the 8.1 nm Cu particles at 300 K is *a* = 3.6107 Å, which is ~0.4% larger than that obtained for the 2 mm Cu ingots.

### 2.2. Intrinsic Magnetic Moment

**Figure 2:**
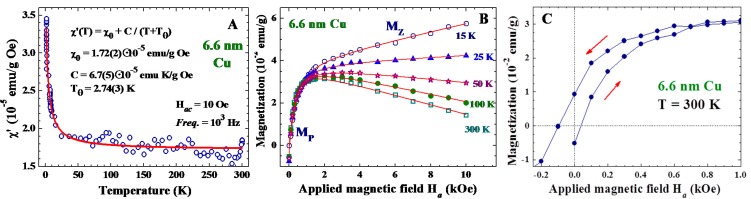
(**A**) Temperature dependencies of the in-phase component χʹ of the ac magnetic susceptibility of 6.6 nm Cu nanoparticle assembly, measured employing a driving ac magnetic field with a root-mean-square strength of 10 Oe and a frequency of 10^3^ Hz. The solid curve indicates the fit to the Curie-Weiss thermal profile; (**B**) isothermal magnetization curves of the 6.6 nm Cu particle assembly, taken in field-increasing loops at five representative temperatures. The solid curves indicate the results of the fit to the field profile discussed in the text; and (**C**) magnetic hysteresis loops of the 6.6 nm Cu assembly at 300 K, revealing a coercive field of 100 Oe and a remanence of 9.4 × 10^−3^ emu/g. The red arrows indicate the directions of the field-changing processes.

It is known that Cu in its bulk form is weakly diamagnetic. Interestingly, the 6.6 nm Cu NPs exhibit paramagnetic responses at all temperatures studied (Figure 2A), showing that the diamagnetic responses from the inner core have been overcome. In addition, the in-phase component of the ac magnetic susceptibility χʹ reduces to an unusually large value at high temperatures. The χʹ(T) can be described as having a Curie-Weiss thermal profile plus a temperature independent response of χ_0_ = 1.72 × 10^−5^ emu/(g Oe)^−1^ (solid curve in Figure 2A). This χʹ(T) was measured without the presence of an applied magnetic field H*_a_*, but revealed thermo-paramagnetic responses to the weak driving ac magnetic field. The appearance of a sizable temperature independent χ_0_ signals the existence of a spontaneous magnetic component in the NPs, which is essentially not affected by the thermal energy even at 300 K.

**Figure 3:**
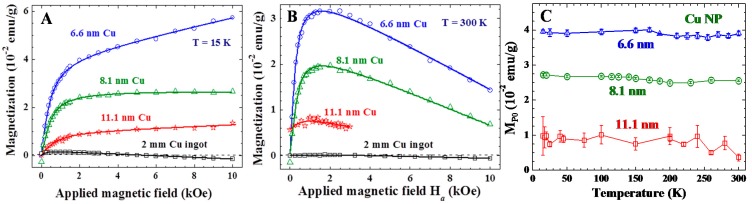
Isothermal M(H*_a_*) curves of the three sets of Cu nanoparticle assemblies together with that of 2 mm Cu ingots at (**A**) 15 K and (**B**) 300 K. The solid curves indicate the results of the fit to the field profile discussed in the text; and (**C**) temperature dependencies of saturation spontaneous magnetization M_P0_ of the three sets of Cu nanoparticle assemblies.

An isolated NP with a spontaneous magnetic moment can be treated as a superspin [33,34], where the magnetic behavior is expressed by a particle magnetic moment of several hundreds or thousands Bohr magnetons. The assembly of loosely packed magnetic NPs is frequently described as a superparamagnetic system that consists of a macroscopic amount of non-interacting superspins [35,36]. The particle moments in the assembly can be more clearly revealed in the isothermal magnetization M(H*_a_*) measurements, where the H*_a_* serves to align the superspins along the field direction. Figure 2B displays the M(H*_a_*) curves of the 6.6 nm Cu NP assembly, taken at five representative temperatures in a field-increasing loop. In the low-H*_a_* regime, M increases rapidly with increasing H*_a_*. This component, marked M_P_, is clearly revealed even at 300 K, without much reduction in the magnitude upon warming from 15 to 300 K. At 15 K, M continues to increase at higher H*_a_*, but with a much reduced rate, showing the appearance of an additional component. This component, marked M_Z_, becomes barely seen at 25 K (Figure 2B). Clearly, M_P_ and M_Z_ are linked to different origins. The linear Lenz diamagnetic component begins to dominate in the high-H*_a_* regime at 50 K (Figure 2B). There are, hence, three components that appear in the magnetization curve: M(H*_a_*) = M_P_ + M_Z_ + χ_D_H*_a_*, where χ_D_ is the Lenz diamagnetic susceptibility. M_P_ may be satisfactorily described by a Langevin profile:

(1) $${\text{M}}_{\text{P}}={\text{M}}_{\text{P0}}\{\text{coth}\left(\frac{{\text{µ}}_{\text{P}}{\text{H}}_{a}}{k_{\text{B}}\text{T}}\right)-\frac{k_{\text{B}}\text{T}}{{\text{µ}}_{\text{P}}{\text{H}}_{a}}\}$$

where M_P0_ indicates the saturation particle magnetization of the assembly, μ_P_ is the average particle moment of the superspins, and *k*_B_ is the Boltzmann’s constant. The Langevin profile for M_P_ may be understood as the magnetic moments of the randomly oriented non-interacting superspins of average particle moment μ_p_ that are being aligned by H*_a_*. The existence of spontaneous particle moments is also revealed in the M(H*_a_*) loops. Magnetic hysteresis with a coercive field of 100 Oe and a remanence of 9.4 × 10^−3^ emu/g is clearly revealed at 300 K (Figure 2C). Although the coercive field and remanence are relatively small, they are, nevertheless, direct evidences of the existence of an intrinsic magnetic moment in the 6.6 nm Cu NP. M_P_ is found to be sensitive to the particle size as well (Figure 3A), but is still visible in the 11.1 nm Cu NPs at 300 K (Figure 3B).

M_Z_ is rarely seen in larger NPs even at 15 K (Figure 3A), indicating that it is sensitive to the size of the NP. The appearance of M_Z_ can originate from the discrete nature of the electron level in NPs, where quantum confinement causes the electronic bands near the Fermi level to split into discrete sub-bands separated by Kubo gaps [15]. Contribution to magnetization from quantum-confined Zeeman split spin polarized states can be expected [26]. Zeeman magnetization of the quantum-confined spins follows a Brillouin profile [37]:

(2) $${\text{M}}_{\text{Z}}({\text{H}}_{a},\text{T})={\text{M}}_{\text{Z0}}\{\frac{2J+1}{2J}\text{coth}\left[\frac{\left(2J+1\right)}{2J}\frac{{\text{gµ}}_{\text{B}}{\text{H}}_{a}}{k_{\text{B}}\text{T}}\right]-\frac{1}{2J}\text{coth}\left(\frac{1}{2J}\frac{{\text{gµ}}_{\text{B}}{\text{H}}_{a}}{k_{\text{B}}\text{T}}\right)\}$$

where M_Z0_ is the induced saturation magnetization, J is the quantum number of total angular momentum, *g* is the Lande g-factor, and µ_B_ is the Bohr magneton. Competition between thermal agitation and field alignment results in a Langevin type of isothermal magnetization curve M_P_(H*_a_*); whereas thermal excitation of the valence and conduction electrons into Zeeman split spin polarized states gives rise to the Zeeman magnetization M_Z_(H*_a_*). Apparently, thermal populations from the down-spin state to the up-spin state at 25 K have nearly decompensated for the H*_a_*-induced Zeeman magnetization in 6.6 nm Cu NPs.

The solid curves in Figure 2 and Figure 3 are the result of the fit of the data to the isothermal field profile discussed above. All the M(H*_a_*) curves observed can be satisfactorily described. The fits obtained assuming *J* ≥ 0.72 for M_Z_ give nonphysical results, leading us to take *J* = 1/2. This signals that it is only the spin magnetic moment that contributes to the Zeeman magnetization, which agrees with that the Kubo bands in Cu NPs are mainly associated with the delocalized conduction electrons. An average particle moment of μ_p_ = 987 μ_B_ is obtained for the superspins of 6.6 nm particles at 15 K. In addition, it is remarkable to see that the saturation particle moments M_P0_ of all three sets of NP assemblies are only slightly reduced upon warming from 15 to 300 K (Figure 3C), showing that the superspin in each NP will persist up to a temperature well above 300 K. The Lenz diamagnetic susceptibility χ_D_ obtained from the fit is −2.41 × 10^−6^ emu/(g Oe)^−1^ for the 6.6 nm Cu NPs at 300 K, which is 22 times larger than the corresponding values of −1.1 × 10^−7^ emu/(g Oe)^−1^ for the 2 mm Cu ingots.

### 2.3. Electronic Charge Redistribution

**Figure 4:**
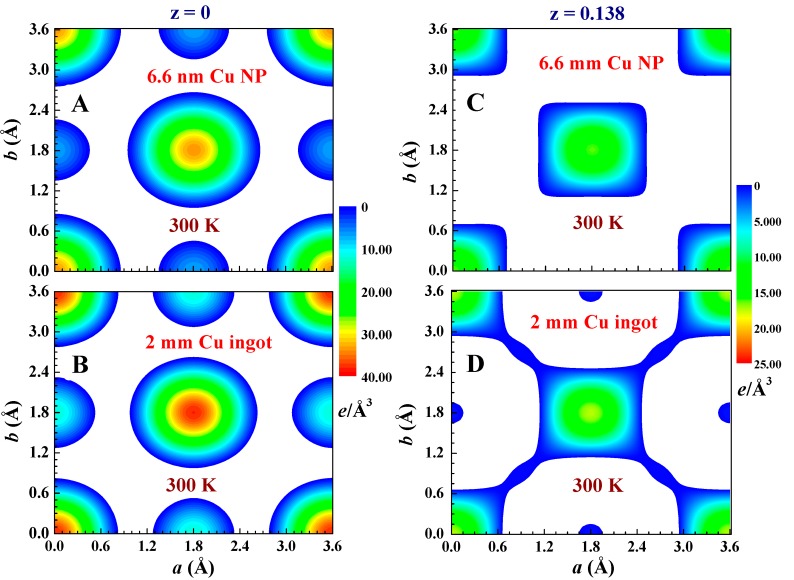
(**A**,**B**) Electronic charge densities in the z = 0 crystallographic plane of the 6.6 nm Cu nanoparticles and 2 mm Cu ingot, as inferred from the X-ray diffraction data; and (**C**,**D**) electronic charge densities in the z = 0.138 crystallographic plane of the 6.6 nm Cu nanoparticles and 2 mm Cu ingot. The color bars are in units of *e*/Å^3^.

It is remarkable to find that the electronic charge distribution of Cu NPs is significantly different from that of Cu ingots. The inner electronic charge densities of the NP are noticeably less intense, while the outer electrons are more extended distributed (Figure 4A,B). In addition, the electron distributions in the NPs are not evenly extended in all crystallographic directions, but reveal less connection with its neighbors along specific directions, such as along the (110) crystallographic direction in the (0, 0, 0.138) lattice plane (Figure 4C,D). These electron density maps were obtained by employing the General Structure Analysis System (GSAS) program, starting with a profile refining the X-ray diffraction pattern, followed by calculation of the inverse Fourier transforms of the structure factors to extract the electron density distribution. The electron density contour map of a specific plane was then obtained by slicing the electron density in the vicinity, including 0.025 Å below and above the plane. The electronic charge density along a specific crystallographic direction could then be obtained by cutting the density map along the selective direction.

**Figure 5:**
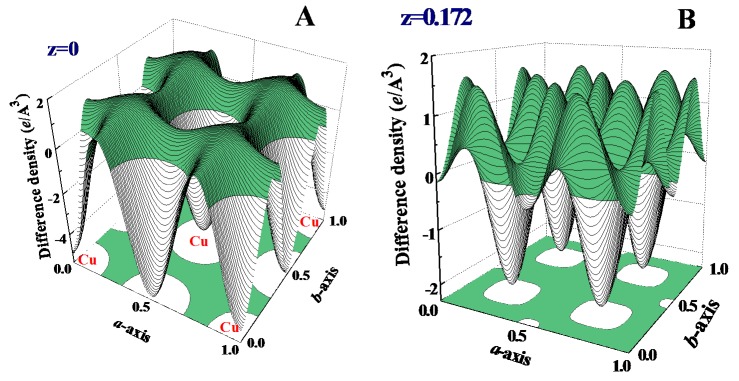
Difference of the electronic charge density between the 6.6 nm Cu assembly and 2 mm Cu ingot on the (**A**) z = 0 and (**B**) z = 0.172 planes. The color-filled regions indicate the positions having a positive difference charge density, where the charge density of the nanoparticles is higher than that of the 2 mm ingot. The projection of the electronic charge density onto the lattice plane is shown at bottom.

It is clear that a reduction of the particle size to the nano-meter scale can cause a significant redistribution of the electronic charge density. These changes are better revealed in the difference density plots, where the electron density of the 2 mm ingot is subtracted from that of the 6.6 nm NP. Such difference density plots for the (0, 0, 0) and (0, 0, 0.172) crystallographic planes are illustrated in Figure 5A,B, respectively. The atomic positions with negative values of difference density represent the locations where electronic charges are less in NP, but redistributed to the positions with positive values. The color-filled regions indicate the positions having a positive difference density. These positions can be more clearly seen in the projection of the difference density onto the lattice plane shown at the bottom of Figure 5. More of the electronic charges in the NPs are found between the two nearest neighbors, but less around the lattice sites in the (0, 0, 0) plane (Figure 5A). The situation is different in the (0, 0, 0.172) plane, where fewer electronic charges in NPs are seen around the center positions of the two nearest neighbors. This spatially uneven change of the electronic charge density over crystallographic directions cannot be associated only with the redistribution of the *s* electrons, but requires the redistribution of the *d* and/or *p* electrons as well.

**Figure 6:**
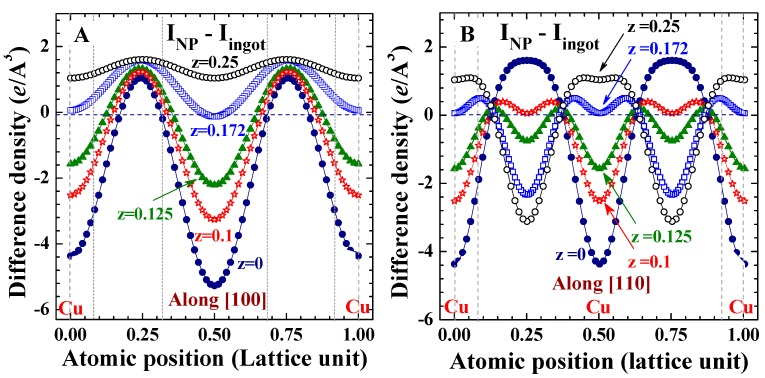
Difference of the electronic charge density between the 6.6 nm Cu assembly and 2 mm Cu ingot along the (**A**) (100) and (**B**) (110) crystallographic directions at z = 0 (filled circles), 0.1 (open stars), 0.125 (filled triangles), 0.172 (open squares), and 0.25 (open circles). The shaded areas indicate the regions of the inner cores.

**Figure 7:**
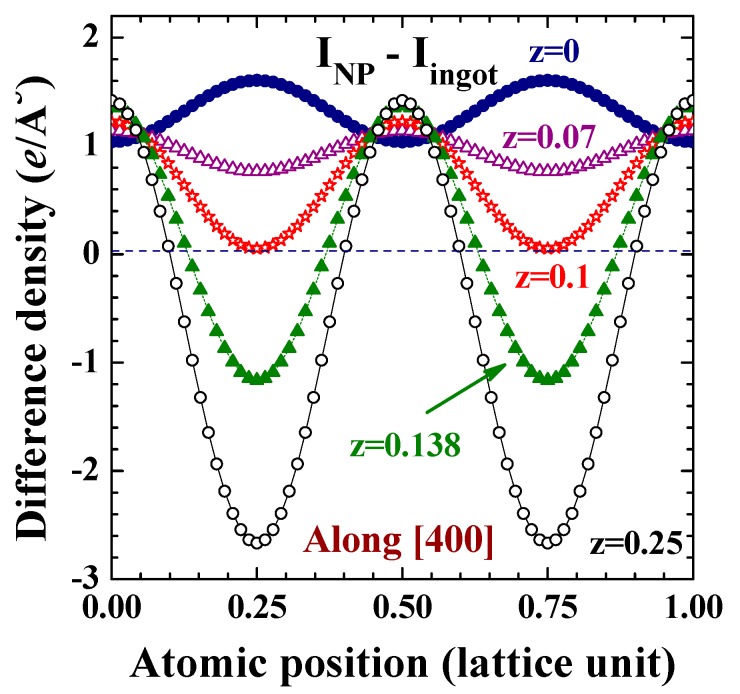
Difference of the electronic charge density between the 6.6 nm Cu assembly and 2 mm Cu ingot along the (400) crystallographic directions at z = 0 (filled circles), 0.07 (open triangles), 0.1 (open stars), 0.138 (filled triangles), and 0.25 (open circles).

It is known that the inner electrons in the core (1*s* to 3*d* electrons) of each Cu ion in a crystallized fcc structure extended from the lattice site to cover a spatial region with a radius of ~7.5% of a lattice unit, with the 3*d* orbital extended to the outmost area of the inner core region and the 3*s* orbital is slightly within. Interestingly, the electron density of the inner core is noticeably lower in the NPs (shaded areas in Figure 6A,B), and this region of less electrons in NPs extends into the 4*s* orbital to reach ~17.5% of a lattice constant (Figure 6A). Beyond this the electronic charge density of the NPs becomes higher, extending only up to ~32.5% of a lattice constant (Figure 6A). Remarkably, reducing the size of Cu to the nanometer scale results in more electrons being distributed in the central regions of the two nearest neighbors (filled circles in Figure 6B), up to a spatial region of ~10% of a lattice constant below and above the lattice plane (open stars in Figure 6B). Further away from this the electron density in the NPs becomes less intense. The shifting of electrons away from closer to the center position of the two nearest neighbors can be better revealed in the difference density plots along the (400) crystallographic direction (Figure 7). It is clear that the spatially-extended distribution of the electronic charge in NPs is not isotopically extended in all crystallographic directions, but rather a portion of electrons shift from specific regions to the others. The redistribution involves not only spherically-distributed 4*s* electrons, but also includes directional 3*d* electrons.

It is very likely that the development of ferromagnetic spin polarization in Cu NPs is the direct result of an electronic charge redistribution that involves 3*d* electrons. The existence of superspin moments is revealed in the isothermal Langevin M(H*_a_*) profiles (Figure 2B and Figure 3A,B), showing that localized 3*d* holes do exist for the development of particle superspin moments. In addition, an enlarged conduction electron density is revealed in the enhanced zero-field Curie-Weiss χʹ(T) profile (Figure 2A). Clearly, the charge redistribution initiated by reducing the particles to nano-sizes involves both the conduction 4*s* electrons and localized 3*d* electrons. This can happen only when the 4*s* and 3*d* bands are energetically close to each other. Band structure calculation [38] for bulk Cu has shown that although the outer bands of Cu can be separated into five narrow 3*d* bands and one broad 4*s* band, at some values of wave vector all six electron bands are energetically closer together, where distinction between 3*d*-band and 4*s*-band levels is not meaningful [27]. An extended 3*d* and 4*s* band mixture accommodating the disruption of lattice periodicity at the surfaces, known as the small size effect, can then be anticipated to give rise to an anisotropic electronic charge redistribution.

## 3. Experimental Section

The X-ray diffraction measurements for structural investigation and particle size determination were performed on a Bruker D8 ADVANCE diffractometer (Bruker Corporation: Billerica, MA, USA), employing the standard reflection geometry. The magnetization and AC magnetic susceptibility measurements were performed with a Physical Property Measurement System (Quantum Design: San Diego, CA, USA), manufactured by Quantum Design, employing the standard setups. The NPs were very loosely packed for these measurements, so that they reveal mainly the magnetic responses from individual NPs without significant contributions from interparticle interactions. To avoid any aggregation that may arise among the NPs, the powder was shaken at 50 Hz for 5 min using a Vortex-Genie Mixer (Scientific Instruments Inc.: Skokie, IL, USA). The NPs (~25 mg each) were packed into a non-magnetic cylindrical holder provided by Quantum Design. The packing fraction *f*, which marks the ratio between the mass densities of the NP assembly in the holder and that of bulk Cu, is used to quantify the average interparticle separation in the assembly. The packing fraction chosen for all NP powders used in the present studies is ~15%, which corresponds to an average interparticle separation, from edge-to-edge, of 5.7 nm. Note that when the NPs are naturally assembled together, the packing fraction of the resultant powder is low. This is understood to be because the assembly consists of very many loosely connected NP aggregates and the NPs within each aggregate are only weakly linked together, so that the spatial filling factor of the NPs and their aggregates is considerably low when they are naturally packed. The holder produces a smooth temperature curve and a background signal that is ~2% of the signal from the samples.

## 4. Conclusions

The present study focuses on identifying the magnetic properties of bare Cu nanoparticles. Three sets of bare Cu nanoparticle assemblies having mean particle diameters of 6.6, 8.1, and 11.1 nm were fabricated employing the gas condensation method, based on a physical process involving the self-nucleation of atoms to form capping free Cu nanoparticles. The Curie-Weiss paramagnetic responses of the Cu nanoparticles to a driving magnetic field were detected at all temperatures, showing the appearance of an extra magnetic component that overcomes the diamagnetic responses from the inner core. This component links to the 4*s* conduction electrons, since the Curie constant C is proportional to the density of the conduction electrons. The isothermal magnetization M(H*_a_*) curves reveal Langevin field profiles, reflecting the alignment of the particle superspins to along the field direction by the H*_a_*. Magnetic hysteresis was clearly revealed even at 300 K, demonstrating the existence of ferromagnetic correlations in the Cu nanoparticles. Saturation magnetization reduces only slightly upon warming from 15 to 300 K, indicating that the superspin moments persist to a much higher temperature. The development of superspin moments in Cu nanoparticles is understood as an extended 3*d* and 4*s* band mixture that causes more electronic charges to be distributed around the center position of the two nearest neighbors.

The general picture established so far for the appearance of magnetic moments in polymer-capped noble nanoparticles is that the strong chemical affinity between the capping molecules and the atom is a prerequisite for triggering electron redistribution. This creates localized *d* holes for ferromagnetism in the surface region. Ferromagnetism is, thus, associated only with the atoms on the surface while the core atoms remain diamagnetic. The present study shows that magnetic moments do develop in bare Cu nanoparticles, and the core atoms contribute to the ferromagnetism as well. It appears that a 3*d* and 4*s* band mixture associated with core atoms is visible in Cu nanoparticles. There are at least three effects that can alter the electron band structure and trigger electron redistribution as particles are reduced to the nano-meter size: firstly, disruption of the lattice periodicity at the surface; secondly, the transfer of electrons from the surface into the core matching the Fermi energy of the two regions; and thirdly, the splitting of electronic bands near the Fermi level into discrete levels triggered by the quantum confined boundary conditions. From the present results, however, it is not feasible to distinguish the contribution from each effect, rather than demonstrate the occurrence of an electronic charge redistribution. Investigations using nanoparticles of various sizes can provide more information on this subject from which to discriminate the contribution from each effect.
